# Dynamics and Potential Impact of the Immune Response to Chronic Myelogenous Leukemia

**DOI:** 10.1371/journal.pcbi.1000095

**Published:** 2008-06-20

**Authors:** Peter S. Kim, Peter P. Lee, Doron Levy

**Affiliations:** 1 Laboratoire des Signaux et Systèmes, Ecole Supérieure d'Electricité, Gif-sur-Yvette, France; 2 Division of Hematology, Department of Medicine, Stanford University, Stanford, California, United States of America; 3 Department of Mathematics and Center for Scientific Computation and Mathematical Modeling (CSCAMM), University of Maryland, College Park, Maryland, United States of America; Utrecht University, Netherlands

## Abstract

Recent mathematical models have been developed to study the dynamics of chronic myelogenous leukemia (CML) under imatinib treatment. None of these models incorporates the anti-leukemia immune response. Recent experimental data show that imatinib treatment may promote the development of anti-leukemia immune responses as patients enter remission. Using these experimental data we develop a mathematical model to gain insights into the dynamics and potential impact of the resulting anti-leukemia immune response on CML. We model the immune response using a system of delay differential equations, where the delay term accounts for the duration of cell division. The mathematical model suggests that anti-leukemia T cell responses may play a critical role in maintaining CML patients in remission under imatinib therapy. Furthermore, it proposes a novel concept of an “optimal load zone” for leukemic cells in which the anti-leukemia immune response is most effective. Imatinib therapy may drive leukemic cell populations to enter and fall below this optimal load zone too rapidly to sustain the anti-leukemia T cell response. As a potential therapeutic strategy, the model shows that vaccination approaches in combination with imatinib therapy may optimally sustain the anti-leukemia T cell response to potentially eradicate residual leukemic cells for a durable cure of CML. The approach presented in this paper accounts for the role of the anti-leukemia specific immune response in the dynamics of CML. By combining experimental data and mathematical models, we demonstrate that persistence of anti-leukemia T cells even at low levels seems to prevent the leukemia from relapsing (for at least 50 months). As a consequence, we hypothesize that anti-leukemia T cell responses may help maintain remission under imatinib therapy. The mathematical model together with the new experimental data imply that there may be a feasible, low-risk, clinical approach to enhancing the effects of imatinib treatment.

## Author Summary

Recent mathematical models have been developed to study the dynamics of chronic myelogenous leukemia (CML) under imatinib treatment. None of these models incorporates the anti-leukemia immune response. Recent experimental data show that imatinib treatment may promote the development of anti-leukemia immune responses as patients enter remission. Using these experimental data, we developed a mathematical model to gain insights into the dynamics and potential impact of the resulting anti-leukemia immune response on CML. The mathematical model suggests that anti-leukemia T cell responses may play a critical role in maintaining CML patients in remission under imatinib therapy. Furthermore, it proposes a novel concept of an “optimal load zone” for leukemic cells in which the anti-leukemia immune response is most effective. Imatinib therapy may drive leukemic cell populations to enter and fall below this optimal load zone too rapidly to sustain the anti-leukemia T cell response. As a potential therapeutic strategy, the model shows that vaccination approaches in combination with imatinib therapy may optimally sustain the anti-leukemia T cell response to potentially eradicate residual leukemic cells for a durable cure of CML.

## Introduction

Chronic myelogenous leukemia (CML) results from the uncontrolled growth of white blood cells due to up-regulation of the abl tyrosine kinase [1]. The standard first-line therapy against CML is imatinib, a molecular-targeted drug that inhibits the abl tyrosine kinase [2]. Under imatinib, nearly all patients attain hematologic remission (HR) [3] and 75% achieve cytogenetic remission (CR) [4]. However, imatinib does not completely eliminate residual leukemia cells and patients inevitably relapse after stopping treatment [4]. We note that for a hematologic remission (also known as complete hematologic response) the following must be present: Platelet count 450,000/*µL*, WBC count <10,000/*µL*, WBC differential: no immature granulocytes and <5% basophils, Spleen nonpalpable. Cytogenetic remission (or response) is defined with the following sub-categories. None: Ph+ cells >95%; Minimal: Ph+ cells 66–95%; Minor: Ph+ cells 36–65%; Partial: Ph+ cells 1–35%; Complete: Ph+ cells 0%.

In this paper, we model the dynamics of T cell responses to CML. Insights gained from this model were used to develop a possible combination between imatinib and immunotherapy, in the form of cancer vaccines, to enhance the efficacy of imatinib treatment and potentially eliminate leukemia for a durable cure.

Various papers have proposed hypotheses concerning the effects of imatinib treatment on leukemia cells from a dynamical systems perspective. In a recent work, Michor *et al.* develop a model for the interaction between leukemia and imatinib [5]. In their model, they assume that leukemia cells differentiate through four stages of their life cycle, beginning with leukemia stem cells. Imatinib functions by reducing the rate at which leukemia cells pass from one stage to the next, causing a rapid drop in the leukemia population. Based on their assumptions and analysis, they propose that leukemia inevitably persists, because imatinib hinders the differentiation of differentiated leukemia cells, but does not affect the leukemia stem cells. In particular, Michor *et al.* hypothesize that there is always a steadily growing population of leukemia stem cells despite imatinib treatment. As a result, based on their model, the leukemia population under imatinib eventually relapses, regardless of whether the model considers imatinib resistance mutations.

In a subsequent paper [6], Roeder *et al.* develop a similar model of CML and imatinib. However, they subdivide the leukemia stem cells into two compartments: proliferating and quiescent cells. Proliferating leukemia stem cells are affected by imatinib, while quiescent leukemia stem cells are not affected. Due to this additional assumption, the leukemia population under imatinib does not relapse without the effects of imatinib resistance mutations. Instead, under imatinib treatment, the leukemia stem cell population restabilizes at lower equilibrium level and does not continue growing as in the Michor model.

Both [5] and [6] propose that imatinib does not eliminate the leukemia stem cell population. Consequently, the papers conclude that imatinib therapy should be combined with an additional treatment that either directly impacts leukemia stem cells or causes leukemia stem cells to become vulnerable to imatinib.

As an alternative approach, Komorova and Wodarz develop a model that focuses on the drug resistance of leukemia cells [7]. In their model, they implicitly assume that imatinib affects all leukemia cells including stem cells and that inevitable relapse is a result of acquired imatinib resistance mutations. Komorova and Wodarz consider the possibility of treating patients with multiple drugs to reduce the probability of any leukemia cell eventually acquiring resistance-mutations to all drugs. They determine that a treatment strategy consisting of three leukemia-targeted drugs of different specificity might have a strong chance of eliminating the disease.

The three approaches discussed above present a variety of hypotheses for the dynamics of imatinib treatment on leukemia cells. These papers also propose potential treatment strategies to enhance the effectiveness of imatinib. However, the difficulty with these treatments is that it is unclear what kind of drug could be used to target leukemia stem cells or what alternative drugs could be used in addition to imatinib for a multiple-drug strategy.

In this paper, we model the anti-leukemia immune response in CML patients on imatinib therapy. Biological insights from the model lead us to propose a novel approach that incorporates the leukemia specific immune response into the mathematical models. We show that the model of Michor *et al.* [5], when extended in time, predicts a relapse approximately three years after the start of treatment. However, a three-year relapse conflicts with clinical observations as patients under imatinib often remain in cytogenetic remission for several years. The models of Roeder *et al.* and Komorova and Wodarz present alternative models that may explain the long-term remission typically observed in patients; however, none of these approaches consider the dynamics and impact of the *immune response* to CML.

Recent experiments by Chen *et al.*, observe that some CML patients under imatinib-induced remission develop a robust but transient anti-leukemia immune response involving both CD4+ and CD8+ T cells [8]. The results of Chen *et al.* extend the findings of Wang *et al.* pertaining to antigen-presenting cells and CD4+ T cells in CML [9]. By developing a model that combines imatinib and immune dynamics, we formulate an alternative hypothesis about how remission is sustained and propose a novel treatment strategy to enhance the effectiveness of imatinib.

The paper is organized as follows. In the Materials and Methods section we develop a mathematical model for the dynamics of CML, imatinib, and the imatinib-induced immune response to CML. This model is written as a system of delay differential equations (DDEs) where the delay accounts for T cell division. As part of the model presentation, we pay considerable attention to discussing the parameter estimates. This discussion is divided into two parts. First we deal with the estimation of the universal parameters, i.e., the parameters for which we assume that their range is identical for all patients. We then proceed to discuss the estimation of the three patient specific parameters. This estimation is done by fitting the simulations of the model to the experimental data from [8].

In the Results section we use simulations of our model to discuss the brief anti-leukemia immune response that occurs during imatinib-induced remission. We hypothesize that the immune response serves to sustain leukemia remission longer than it would last otherwise. At the same time, we do point out that this immune response dies off too quickly to be effective at completely eliminating CML.

The work of [8] has also indicated that when an anti-leukemia immune response is not detectable, it can be re-stimulated by in vitro incubation with irradiated autologous leukemia cells or lysates (available from cryopreserved blood from the patient before imatinib therapy). We hypothesize that a similar stimulation of the anti-leukemia immune response can be also obtained in-vivo. We refer to such a procedure as a “cancer vaccine”. We modify our mathematical model to include terms that account for the cancer vaccines. Through mathematical simulations of this new model we show that if indeed a similar response to what was seen in vitro can be also obtained in patients, one can possibly use properly timed vaccines to develop an anti-leukemia response that will be of sufficient magnitude and duration to eradicate all residual leukemia cells. The timing of the vaccine and the doses are tailored to the specific measurable parameters of the immune response of each patient. We study the number of vaccines, their doses, and their timing. We also study the sensitivity of the model to the patient specific parameters. Comments on various aspects of the proposed treatment strategy are provided in the concluding Discussion section.

## Materials and Methods

### A Mathematical Model of the Immune Response to CML

In [8] Chen *et al.* conducted an experimental study involving fourteen patients under imatinib treatment. During the course of treatment, they conducted IFN-*γ* ELISPOT analysis at multiple time points to measure the evolution of the anti-leukemia T cell responses of each patient. All patients achieved HR within 1–3 months. Ten patients achieved complete CR, and 4 achieved major CR. All patients also achieved at least major molecular responses, and sustained molecular as well as cytogenetic responses over time (up to 60 months), except patient 9 (P9), who relapsed after 3 years, and P13, who relapsed after 4 years (after stopping treatment due to imatinib intolerance). We note that a complete molecular response is when the BCR-ABL transcript is non-detectable and non-quantifiable. A major molecular response is defined as BCR-ABL/control gene ratio 0.001.

To study the dynamics of the imatinib-induced immune response, we formulate a mathematical model for leukemia cells and anti-leukemia T cells. The leukemia growth and the response to imatinib follows [5] to which we add interactions with anti-leukemia T cells. Leukemia cells may be killed by interactions with T cells. Also, T cells interacting with leukemia cells may be stimulated to proliferate or to become anergic and die. The T cell interactions are modeled in the same way as in our previous paper [10].

The mathematical model is formulated as a system of DDEs as follows:
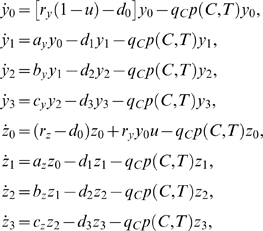
(1)

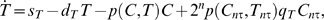
(2)where
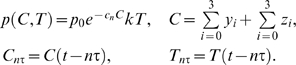



**Figure 1:**
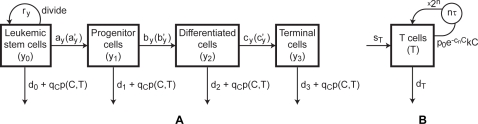
A State diagram for the model in Equations 2 and 3. (A) Cancer cells. The parameters *a_y_*, *b_y_*, *c_y_* correspond to the rates of differentiation of leukemia cells without imatinib treatment, whereas the parameters 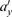, 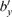, 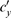 correspond to the rates of differentiation under imatinib treatment. In the case of imatinib-resistant cancer cells, the growth and differentiation rates in the diagram are replaced by *r_z_a_z_*, *b_z_*, *c_z_*. (B) T cells.

A state diagram that corresponds to Equations 1 and 2 is shown in Figure 1. The system of Equation 1 is a modification of the model of [5] for which in each question we added a term that accounts for the death of leukemic cells as a result of an interaction with T cells. The variables *y*_0_, *y*_1_, *y*_2_, and *y*_3_ denote the concentrations of leukemia hematopoietic stem cells (SC), progenitors (PC), differentiated cells (DC), and terminally differentiated cells (TC) without resistance mutations to imatinib. The variables *z*_0_, *z*_1_, *z*_2_, and *z*_3_ denote the respective concentrations of leukemia cells with resistance mutations. The rate constants *a*, *b*, and *c* are given with indices corresponding to non-resistant and resistant leukemia populations. The death rates of the four cell categories are given by *d*_0_, *d*_1_, *d*_2_, and *d*_3_, respectively. The constant *u* is the rate of resistance mutation per cell division.

The variable *C* denotes the total concentration of all leukemia cells with and without resistance mutations. The variable *T* denotes the concentration of anti-leukemia T cells. The final terms in each of the equations in Equation 1 are of the form 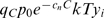 (or 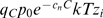). We assume the law of mass action, stating that two cell populations interact at a rate proportional to the product of their concentrations. Hence, the component *kTy_i_* (or *kTz_i_*) is the rate of interaction between T cells and the leukemia subpopulation *y_i_* (or *z_i_*) where *k* is the kinetic coefficient.

The coefficient *p*_0_ is the probability that a T cell engages the cancer cell upon interaction, and *q_c_* is the probability that the cancer cell dies from the T cell response. Furthermore, leukemia cells suppress anti-leukemia immune responses, and while the precise mechanism is unknown, we assume that the level of down-regulation depends on the current cancer population. In particular, we model the probability that a T cell engages a cancer cell decays exponentially as a function of the cancer concentration, i.e., the probability of a productive T cell interaction with a cancer cell is 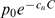 where *c_n_* is the rate of exponential decay due to negative pressure.

It is now well established that cancer suppresses the host immune system in various ways [11]. Leukemia is particularly immunosuppressive as leukemic cells grow within the bone marrow, and can directly suppress both growth and function of normal blood cells. As such, leukemia patients are known to be at higher risk for infections and other cancers [12],[13]. While the mechanisms are varied, we recently showed that cancer patients may have a defect in the interferon signaling pathway [14]. Interferon is an important cytokine in driving immune responses.

In Equation 2, *s_T_* denotes the constant supply rate of T cells into the system from stem cells. The second term is the natural death rate of T cells. The third term is the rate at which T cells engage leukemia cells and commit to *n* rounds of division. The final term represents the population increase due to *n* divisions of stimulated T cells where *τ* is the average duration of one division, and *C_n_*_*τ*_ and *T_n_*_*τ*_ are the time delayed cancer and T cell concentrations respectively. The coefficient *q_T_* is the probability that a T cell survives the encounter with an activated leukemia cell.

The method of modeling T cell proliferation in Equation 2 is similar to what we have previously used in [10]. Once a T cell is stimulated, it exits the collection of interacting T cells and reenters the system *n* *τ* time units later after *n* divisions. This approach ensures that the T cell population does not double faster than once every *n* *τ* days. It is an alternative to using the Michaelis-Menten expression or other saturating terms.

### Parameter Estimates

A considerable amount of effort is devoted to estimating the parameters that appear in our mathematical model (Equations 1 and 2). The discussion is divided into two parts. First, we present the methods for estimating the universal parameters, i.e., the parameters we assume have ranges of values that are similar for all patients. Following the work of [5] we assume that the time-dynamics of cancer is universal, i.e., we describe the evolution of the cancer cells in their various stages of development using parameters that are assumed to be identical for all patients. Clearly, there is no reason to believe that the dynamics of cancer is identical for all patients (as commonly done in mathematical models). Nevertheless, it does serve, in our case, as a way of simplifying the computations in addition to a way to connect between our work and previous works.

We then proceed to describe the methods we used for estimating the remaining three model parameters. These parameters characterize the individual immune response. Consequently they are allowed to vary from patient to patient.

#### Universal parameters

The values of the parameters pertaining to the growth, differentiation, and mutation rates of leukemia cells are taken from [5] without modification. These parameters are *r_y_*, *a_y_*, *b_y_*, *c_y_*, *r_z_*, 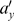, 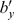, 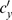, *a_z_*, *b_z_*, *c_z_*, and *u*. The death rates from [5] correspond to the natural death rates of the leukemia populations under imatinib. However, in our model, we distinguish between the natural death rate of leukemia and the death rate due to the cytotoxic T cell response. Hence, our natural death rates, *d_i_*, should be a fraction, *λ*, of the combined death rates estimated in [5].

Determining what fraction *λ* of the leukemia death rates from [5] result from non-immune versus immune causes is difficult and requires some assumptions. First, we assume that *λ* is greater than 0.5, so that the anti-leukemia immune response contributes to less than half of the decline in leukemia under imatinib treatment. Due to the lack of data on *λ* we set it as *λ* = 0.75. A discussion on the sensitivity of the results to the choice of *λ* will follow.

For the kinetic coefficient *k*, we use the same value of 1 (*k*/*µL*)^−1^ day^−1^ which was originally drawn in [10] from the rate constant of virus elimination in [15]. For T cell-cancer interactions, we apply the following assumptions from [10]: 20% of the time nothing happens, and both cells survive and depart; 20% of the time cancer lives, and the T cell becomes anergic or dysfunctional; 40% of the time cancer dies, and the T cell survives and moves on; 20% of the time both cancer and the T cell die. From these assumptions, we deduce that the probability of any sort of interaction is *p*_0_ = 0.8, the probability of cancer dying is *p*_0_ *q_C_* = 0.6, and the probability of a T cell surviving is *p*_0_ *q_T_* = 0.4. Hence, *q_C_* = 0.6/0.8 = 0.75 and *q_T_* = 0.4/0.8 = 0.5.

In [15], Luzyanina *et al.* estimate that T cell divisions take between 0.4 to 2 days, and their best fit estimate is 0.6 days. Also, Janeway estimates that primed T cells divide 2 to 4 times per day [16], which corresponds to a duration of 0.25 to 0.5 days. Combining these sources, we conclude that T cell divisions take between 0.25 and 2 days. Since the anti-leukemia T cells are emerging from an environment of immune down-regulation, we assume they divide at the more conservative rate of one division per day.

A summary of the estimated parameters is provided in Table 1.

**Table 1:** Estimates of universal parameters.

Parameter	Description	Estimate	Source
*λ*	Fractional adjustment constant	0.75	Estimate
*d*_0_	SC death rate	0.003 *λ*/day	[5]
*d*_1_	PC death rate	0.008*λ*	
*d*_2_	DC death rate	0.05*λ*	
*d*_3_	TC death rate	*λ*	
*r_y_*	Growth rate for nonresistant cells	0.008/day	[5]
*a_y_*	Rates without imatinib treatment	1.6	
*b_y_*		10	
*c_y_*		100	
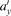	Rates during imatinib treatment	*a_y_*/100	[5]
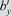		*b_y_*/750	
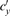		*c_y_*	
*r_z_*	Growth rate for resistant cells	0.023/day	[5]
*a_z_*	Rates for resistant cells	*a_y_*	
*b_z_*		*b_y_*	
*c_z_*		*c_y_*	
*u*	Mutation rate per division	4×10^−8^/division	
*K*	Kinetic coefficient	1 (*k*/*µL*)^−1^ day^−1^	[10],[15]
*p*_0_	Prob. T cell engages cancer cell	0.8	[21]
*q_C_*	Prob. cancer cell dies from encounter	0.75	
*q_T_*	Prob. T cell survives encounter	0.5	
*τ*	Duration of one T cell division	1 day	[15],[16]

#### Patient-Dependent Parameters

The data from [8] for three patients, P1, P4, and P12, each consists of at least five time points per patient. Hence, we focus on these patients when fitting the model to patient data. Tables 2–3 summarize the data from [8] for P1, P4, and P12. Since the duration and the magnitude of the immune responses vary greatly across the three patients, we fit the parameters *s_T_*, *d_T_*, *c_n_*, *n*, *y*_0_(0) to each patient independently and do not attempt to come up with universal estimates of these values. These five parameters denote the supply rate of anti-leukemia T cells, the death rate of anti-leukemia T cells, the level of immune down-regulation by leukemia cells, the average number of T cell divisions upon stimulation, and the initial concentration of leukemia stem cells, respectively.

**Table 2:** Pre-treatment leukemia load.

Patient	P1	P4	P12
Pre-treatment leukemia load (*k*/*µL*)	73.0	23.1	116.8

**Table 3:** Patient data from ELISPOT assay from [8] for P1, P4, and P12.

P1	Time (months)	0	5	30	35	46			
	SFCs/well	3	29	25	25	9			
P4	Time (months)	0	6	9	18	24	32	34	42
	SFCs/well	1	16.5	33	30	26	11	15	12
P12	Time (months)	0	2	5	9	13	15	24	30
	SFCs/well	11	42	39	71	36.5	43	5	6

**Figure 2:**
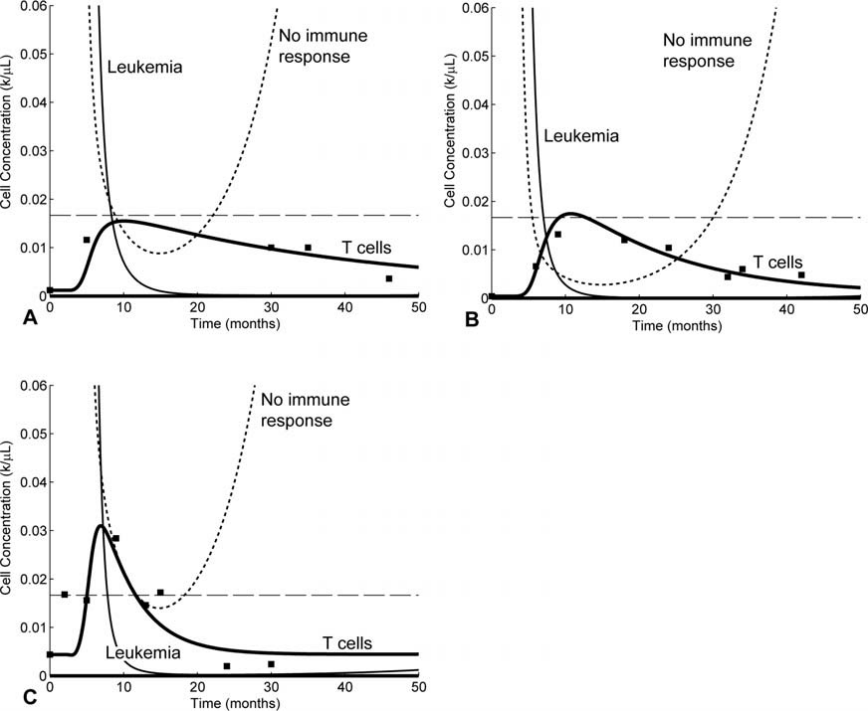
Model solutions fit to data measurements for 3 patients. (A) P1, (B) P4, and (C) P12. The measurements of SFCs/well from [8] are scaled down by 2500 to show relative magnitudes and are shown as black squares. The dashed lines show the approximate level of complete cytogenetic remission. “No immune response” correspond to the predictions of [5]. “Leukemia” correspond to the results of our model (Equations 1 and 2). The “T cells” curve is obtained with our model after fitting the parameters to the experimental data (shown in blank squares).

SFCs/well for leukemia bearing+remission PBMCs. The measurement for time 0 corresponds to pre-treatment leukemia bearing PBMCs. (See Figure 2 for plots of the data points.)

Since even for these three patients only few data points are available, we do not apply a formal method to fit the five patient-dependent parameters to the data. Rather, we use certain features of the data sets, such as the peak height of the T cell response, to estimate the patient-dependent parameters.

We use known information from the literature to determine reasonable ranges for *n* and *d_T_*. To determine an upper bound for the average number of T cell divisions, *n*, we consider that when naïve CD8+ T cells are primed for the first time, they go through several cycles of division. An analysis of experimental data by Antia *et al.* showed that stimulation of naïve CD8+ cells result with up to 8 divisions in vitro [17]. In addition, Janeway estimates that the proliferation of primed CD8+ cells leads to about 10^3^ daughter cells [16], which implies about 10 divisions. Primed CD8+ T cells continue to divide as long as they receive stimulus, but not as many times as during the initial stimulation. Hence, we conclude that primed CD8+ T cells divide fewer than 10 times and most likely fewer than 8 times per stimulation.

To estimate the range of the T cell death rate, *d_T_*, we consider the observations and calculations from [18] that primed CD4+ T cells peak nine days after stimulation, initially die with a half-life of 3 days, and slow down to a half-life of 35 days, eight days after the peak of the response. These numbers yield an initial death rate of 0.23/day and an eventual death rate of 0.02/day. In addition, in [18] it is estimated that primed CD8+ T cells die with a half-life of 1.7 days, yielding a death rate of about 0.4/day. The half-lives of memory CD4+ and CD8+ T cells are much higher, i.e. 500 days to lifelong respectively. Since we are looking at data points that were measured over several years, most of the lingering T cells in the anti-leukemia response are probably CD4+ effector cells or memory CD4+ and CD8+ cells. Since we are examining time-scales of several months to a few years, for convenience, we assume that the T cell death rate is constant at 0.02/day or lower and do not take into account the biphasic switch that probably occurs around seventeen days after the beginning of the immune response.

The characteristics of the five patient-dependent parameters are summarized in Table 4.

**Table 4:** Estimated ranges of patient-dependent parameters.

Parameter	Description	Estimate	Source
*n*	Average number of T cell divisions	1<*n*<8	[17]
*d_T_*	Anti-leukemia T cell death rate	<0.02/day	[18]
*s_T_*	Anti-leukemia T cell supply rate	? (*k*/*µL*)/day	Based on *d_T_* and patient data
*c_n_*	Decay rate of immune responsivity	? (*k*/*µL*)^−1^	Based on patient data
*y*_0_(0)	Initial concentration of leukemia stem cells	? (*k*/*µL*)	Based on patient data

The initial concentration of leukemia stem cells, *y*_0_(0), is the most straightforward parameter to estimate, since its value can be derived directly from the initial leukemia load measured in [8].

If we assume that all populations start in their steady states, we can calculate the initial concentrations of all leukemia cell compartments in terms of *y*_0_(0) and the universal parameters given in Table 1. (Likewise, we can calculate the initial concentration of T cells in terms of the T cell supply and death rates.)

If we assume that there are no resistant cells at the start of treatment, the initial concentration of imatinib resistant stem cells is 0. Note that Michor *et al.* also consider a scenario, in which the initial resistant stem cell count is 10 cells [5]. Assuming that an average person has 6 L of blood, this initial count corresponds to an initial concentration of 10/6 *L*∼10^−9^ *k*/*µL*. Clearly, this is a very crude estimate as the leukemic cells are distributed within the bone marrow, spleen, and blood. However, as will be shown in the sensitivity study below, the initial concentration plays a rather limited role in the emerging dynamics, and thus even such a crude estimate will suffice. See Table 5 for a list of initial concentrations.

**Table 5:** Initial concentrations.

Population	Value (*k*/*µL*)	Reason
*y*_0_(0)	?	Determined by patient data
*y*_1_(0)	*a_y_y*_0_/*d*_1_	Steady state
*y*_2_(0)	*b_y_y*_1_/*d*_2_	Steady state
*y*_3_(0)	*c_y_y*_2_/*d*_3_	Steady state
*z*_0_(0)	0 or 10^−9^	Correspond to values in [5]
*z*_1_(0)	*a_z_z*_0_/*d*_1_	Steady state
*z*_2_(0)	*b_z_z*_1_/*d*_2_	Steady state
*z*_3_(0)	*c_z_z*_2_/*d*_3_	Steady state
*Y*(0)	*s_T_*/*d_T_*	Steady state

To calculate *y*_0_(0), we set the pre-treatment leukemia loads listed in Table 2 equal to the expression for the total initial leukemia concentration, 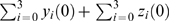, and solve for *y*_0_(0).

The T cell death rate, *d_T_*, is estimated from the rate of decline of the anti-leukemia T cell populations after their peak. Hence, the last three data points for P1, the last five data points for P4, and the last five data points for P12 are used to estimate the rate of T cell death *d_T_*.

If we assume that the T cell population is at steady state before treatment, the concentration of anti-leukemia T cells at time 0 is *s_T_*/*d_T_*. By setting this ratio equal to the initial T cell concentrations obtained from the data in Table 3, we can determine *s_T_* in terms of *d_T_*.

The rate *c_n_* of the decay of the immune response due to negative pressure is difficult to estimate. However, the value of *c_n_* affects the number of T cells that are stimulated during the course of imatinib treatment and how soon T cell expansion initiates. Specifically, we can use the data points before the T cell peak to estimate the time of initiation of the anti-leukemia T cell response for each patient. From the data, it is apparent that the T cell response does not initiate immediately, indicating a lingering immunosuppressive effect from the leukemia cells. We assume that the T cell responses start approximately 2.5, 3, and 2 months after the start of imatinib treatment for patients P1, P4, and P12, respectively.

Given the T cell death rate *d_T_*, we can determine the range of cancer concentrations where the T cell growth rate, 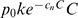, exceeds the T cell death rate, *d_T_*. Before the T cell response starts, the leukemia concentration falls solely based on its natural death rate, since there is no active T cell response. Thus, we can further determine the time that the cancer concentration first reaches the point where the T cell growth rate exceeds the T cell death rate. Hence, we can approximate the value of *c_n_* that causes the T cell responses of P1, P4, and P12 to begin expanding around months 2.5, 3, and 2, respectively. We examine this idea more thoroughly when we introduce the “optimal load zone” for T cell stimulation.

The remaining parameter *n*, which represents the average number of T cell divisions per stimulation, is estimated by matching the results of the simulation to the data points. In particular, the peak height of the T cell response is a strong indicator of the value of *n*, since higher *n* lead to higher T cell peaks.

To fit the patient-dependent parameters, we convert the data from [8] into units of concentration, namely thousands of cells per microliter (*k*/*µL*). The data in [8] is originally given in SFCs/well and 10^5^ PBMCs were used in each well. However, only a fraction of the PBMCs are T cells, and measurements of TNF-*α* and IFN-*γ* in [8] imply that the standard procedure of measuring the IFN-*γ* response using the ELISPOT assay may underestimate the strength of a T cell response. Due to these uncertainties, we assume the measurements from the ELISPOT assay indicate relative magnitudes among T cell responses at various time points, but we do not convert the SFCs/well measurements directly into units of concentration (*k*/*µL*).

In [8], Chen *et al.* conducted TNF-*α* and IFN-*γ* ELISPOT analyses to measure T cell activity. From this data (in particular the data of patient P4), it is seen that roughly 4% of CD4+ T cells and 1% of CD8+ respond to leukemia at the peak of the T cell response. Hence, we scale the ELISPOT data down by 2500 to obtain T cell concentrations. This corresponds to about 1% of T cells from P4 responding to leukemia at the peak of the response. Furthermore, we use the scaled values for the initial ELISPOT measurements at time 0 to set the steady state T cell concentration, *s_T_*/*d_T_*. The cancer-related parameters are given in Table 1. For our first study, we assume that there are no resistance mutations, so we set the mutation rate, *u*, and the initial concentration of imatinib-resistant stem cells, *z*_0_(0), to 0. The remaining parameters for each of the three patients are given in Table 6.

**Table 6:** The parameters for Figure 2.

	Pre-treatment leukemia load→*y*_0_(0)	*n*	*d_T_*	*s_T_*	*c_n_*
P1	73→7.6×10^−6^	1.2	0.001	1.2×10^−6^	1
P4	23.1→2.4×10^−6^	2.2	0.0022	9×10^−7^	7
P12	116.8→1.2×10^−5^	1.17	0.007	3.08×10^−5^	0.8

For any given *d_T_*, *s_T_* is chosen such that the steady state T cell concentration, *s_T_*/*d_T_*, coincides with the ELISPOT measurement at time 0 (scaled by 2500 as in Figure 2).

## Results

### Imatinib-Induced Immune Dynamics

Graphs of the solutions of the mathematical model that correspond to patients P1, P4, P12, along with the measured data points are displayed in Figure 2. The cases labeled “no immune response” in Figure 2 are taken from [5] and correspond to setting the T cell concentrations in Equation 1 to 0, i.e., without the immune response. In comparison to the no-immune-response cases, the T cell response contributes to driving the leukemia population lower than with imatinib alone. Furthermore, the persistence of anti-leukemia T cells at low levels keeps the leukemia population from relapsing for up to several years, whereas in the no-immune-response cases, cancer rebounds are noticeable after 15 to 24 months (see Figure 2).

We estimate the approximate concentration corresponding to complete cytogenetic remission, based on [19]. According to [19], there are 10^12^ leukemia cells prior to imatinib treatment. As a general medical assumption, there are three layers of remission, hematological, cytogenetic, and molecular, and each layer corresponds to a 2 log, or 100-fold, difference from the previous one. Hence, hematological remission corresponds to roughly 10^10^ cells, and cytogenetic remission corresponds to roughly 10^8^ cells. If the average person has 6 l of blood, cytogenetic remission corresponds to a blood concentration of 10^8^/6 l = 1/60 *k*/*µL*. The cytogenetic remission level is shown as dashed lines in Figure 2.

Regarding the no-immune-response case, Michor *et al.* demonstrate that imatinib significantly reduces the populations of differentiated leukemia cells, but does not eliminate leukemia stem cells [5]. As a result, the leukemia population decreases rapidly at the beginning of treatment, while the stem cell population continues to rise exponentially at a much slower rate of *r_y_* – *d*_0_. This phenomenon occurs even in the absence of resistance mutations, making an eventual relapse unavoidable.

On the other hand, our model including the anti-leukemia T cell response predicts a substantially slower relapse and provides a fit to the immunological data. Hence, it is possible that a combination of imatinib and an immune response keeps the leukemia population under control and allows patients to remain in cytogenetic remission for several years. Indeed, the model predicts that the patients remain in cytogenetic remission beyond month 50.

In all three patients, the leukemia cells are not eliminated completely by imatinib treatment. In fact, the lowest concentrations obtained by the cancer populations in Figure 2 for P1, P4, and P12, are 1.3×10^−4^, 7.8×10^−5^, and 2.2×10^−4^ *k*/*µL*,respectively, which correspond to half a million to a million cells remaining in the body, assuming that an average person has 6 L of blood. As can be observed in Figure 2C, it seems that leukemia starts increasing again about 24 months after the start of treatment. This observation stresses an important point, namely that our model does not predict that CML is eliminated by imatinib treatment alone. It does, however, predict that it takes significantly more time for the disease to relapse (when compared with the Michor model).

Nonetheless, leukemia drops to such a low level that the T cells are no longer stimulated and begin to contract. As a result, the immune response does not expand sufficiently to eliminate the leukemia cells. Unfortunately, although imatinib drives the cancer population to low levels, it does not eliminate the leukemia stem cells [5]. Hence, the low population of leukemia stem cells remain below immune surveillance and out of reach of imatinib, escaping complete elimination.

**Figure 3:**
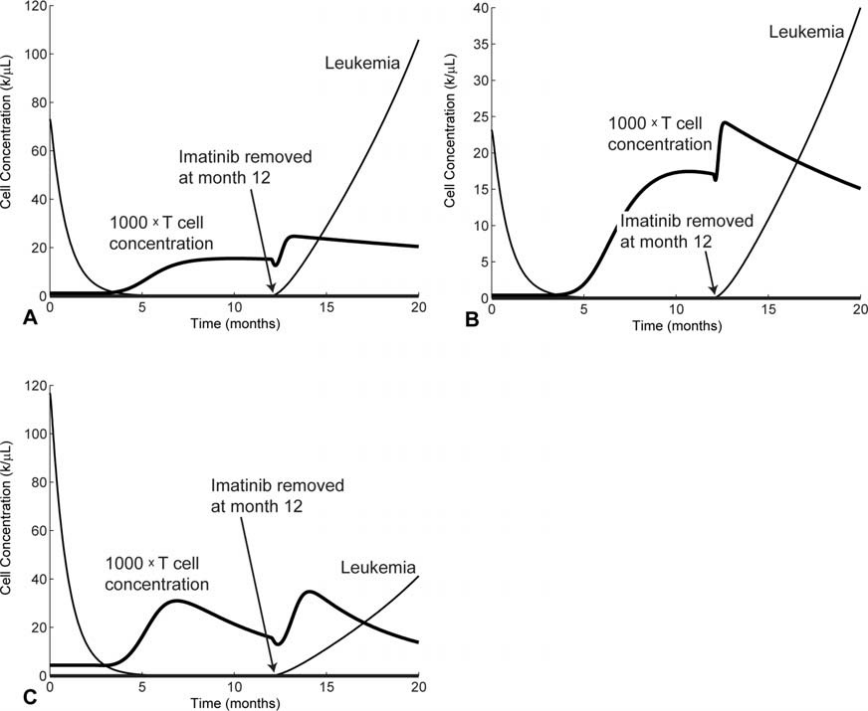
A predicted relapse when imatinib is removed at month 12. The T cell response is never sufficient without imatinib and the removal of imatinib leads to full relapse. (A) P1. (B) P4. (C) P12.

**Figure 4:**
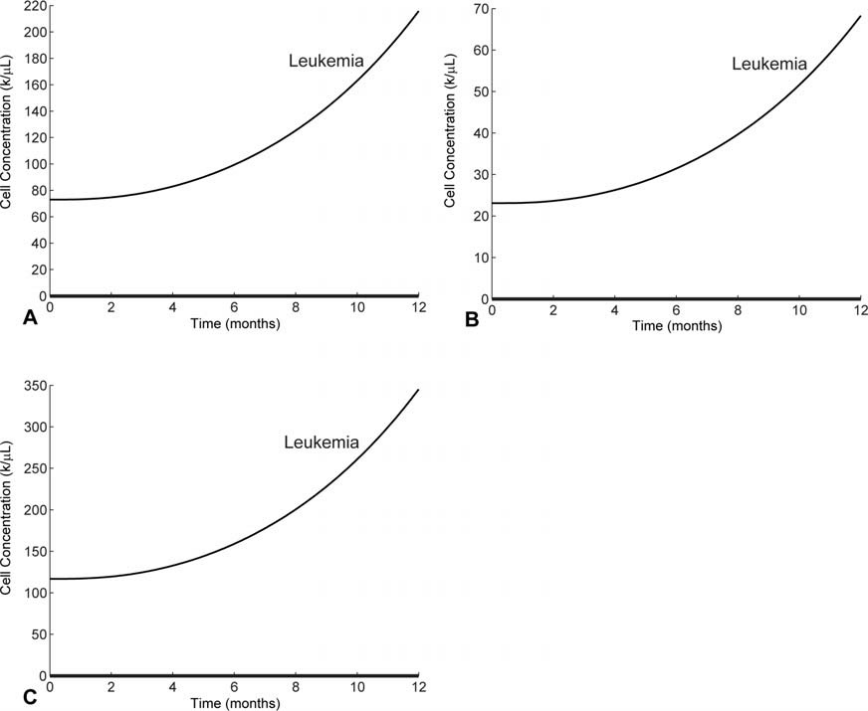
Solving the model equations without an imatinib treatment. The T cell responses are fully suppressed and stay flat at their steady state concentrations while cancer grows rapidly. (A) P1. (B) P4. (C) P12.

In Figure 3 we show simulations for the three patients that demonstrate what happens when the imatinib treatment is stopped at month 12. Similar results are observed for all three patients. The removal of imatinib leads to a resurgence of the leukemia population which causes an initial increase in the T cell response; however, the T cell response is never strong enough to overcome the rapidly growing leukemia population. This result is consistent with clinical observations that patients taken off imatinib invariably relapse [5]. For the purposes of this paper, we assume that the patients are always treated by imatinib. The strong immune responses in Figure 3 are induced by imatinib. Indeed, in the absence of any imatinib treatment, no immune response initiates, a scenario that is shown in Figure 4.

**Figure 5:**
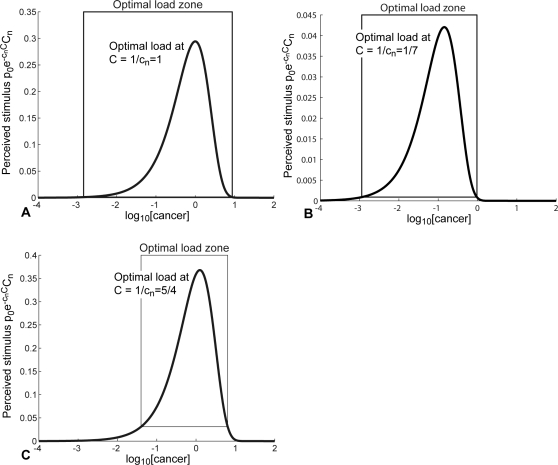
Stimulation levels of anti-leukemia T cells versus logs of the cancer concentrations. Optimal loads are the cancer concentrations *C* for which the perceived stimulus, 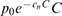, is maximized. Optimal load zones are the range of leukemic concentrations where the T cell stimulation rate is faster than the T cell death rate, i.e. 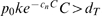. (A) P1. (B) P4. (C) P12.

We would now like to further elaborate on the various aspects regarding the stimulation of the immune response as reflected in our model (Equations 1 and 2). From Equation 2, the balance between immune down-regulation and T cell stimulation by leukemia cells is given by the term 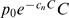. Hence, the optimal level of T cell stimulation occurs at *C* = 1/*c_n_*. We define the *optimal load zone* to be the range of leukemic concentrations where the T cell stimulation rate is faster than the T cell death rate, i.e., 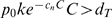, where *k* is the mass-action coefficient and *d_T_* is the T cell death rate. Figure 5 shows the optimal load zones and stimulus levels of T cells as functions of the leukemia concentrations for the three patients. The anti-leukemia T cell populations begin expanding when the leukemia concentration drops into the optimal load zone and begin contracting when the leukemia concentration drops below the optimal load zone.

Figure 5 shows that if the cancer concentrations grow beyond approximately 10^1^ (for the three patients) the perceived stimulus is so low that the anti-leukemia T cell response begins to contract, allowing the cancer population to expand more rapidly. The expanding cancer population then further suppresses the T cell response, leading to an uncontrolled relapse. Hence, we can say that the relapses in Figure 3 are complete, and the immune responses do not recover.

The level of immune down-regulation, *c_n_*, by leukemia cells is a key parameter that governs how well the immune response can function against the relapse following the removal of imatinib (see Figure 3). If *c_n_* is high, the leukemia provides less stimulus for the T cells and passes through the optimal load zone faster during relapse. This makes it less likely for T cells to be able to multiply to sufficient levels to hinder the growth of cancer. However, if *c_n_* is low enough, it is possible that the T cells have proliferated enough during the first imatinib-induced response to stall cancer growth, provided that imatinib is removed near the time of the T cell peak. Nonetheless, as we have seen in Figure 4, the immune response against a relapsing cancer population is expected to be ineffective. The quantity *c_n_*, which varies from patient to patient, measures the ability of cancer cells to down-regulate the anti-cancer immune response. It is unknown whether all leukemias exert roughly the same negative pressure or whether this parameter can vary widely.

### A Combined Treatment Strategy

As shown previously at the beginning of treatment, imatinib causes the leukemia population to drop into the optimal load zone, stimulating an immune response. However, under continued treatment, the leukemia population quickly drops below the optimal load zone, and the T cell population contracts due to lack of stimulus. A strategy to maintain the leukemia population within the optimal load zone or to surrogately stimulate anti-leukemia T cells may help in driving the leukemia population to zero.

The experimental results of [8] suggest that autologous leukemia cells may be collected from a patient, inactivated, and strategically reintroduced to enhance the anti-leukemia T cell response. Ideally, these vaccinations stimulate the immune system enough to drive the residual leukemia population to zero. We assume that imatinib is administered throughout the entire course of the therapy.

To study the feasibility of this approach, we introduce inactivated leukemia cells into our model (Equations 1 and 2). Inactivated leukemia cells (whose number is denoted by *V*) die or decay at a constant rate *d_V_* and are supplied into the system in vaccination boosts at rate *s_V_*(*t*). All leukemia cells may be killed by interactions with T cells. T cells interacting with leukemia cells may be stimulated to proliferate or become dead or anergic. These interactions are also modeled in the same way as in [10]. We modify the original model by adding Equation 3 and replacing Equation 2 with Equation 4:
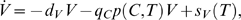
(3)

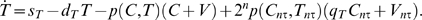
(4)Here, *V* denotes the concentration of inactivated leukemia cells and *V_n_*_*τ*_ = *V*(*t*−*n* *τ*). We assume that T cells always survive encounters with inactivated leukemia cells, so that there is no coefficient *q_T_* before the variable *V_n_*_*τ*_ in Equation 4.

The leukemia cells used in vaccinations can be inactivated via irradiation. Since they are in the process of dying, we estimate that they do not survive much longer than 24 to 72 hours, so we set the decay rate *d_V_* = 0.35, which corresponds to a half life of 2 days. The supply rate *s_V_*(*t*) of inactivated leukemia cells will be 0 except when the vaccination is being delivered. During vaccination, we estimate that a total quantity *q_V_* of inactivated leukemia cells is delivered at rate 100*q_V_* for a duration of 0.01 days, which is slightly under 15 minutes. The parameters related to inactivated leukemia cells are given in Table 7.

**Table 7:** Parameter estimates for inactivated leukemia cells.

Parameter	Description	Estimate
*V*(0)		Presumably vaccinations begin after time 0
*d_V_*	Decay rate of inactivated leukemia cells	0.35/day
*q_V_*	Vaccination dosage	To be optimized
*t_V_*	Duration of delivery	0.01 day
*s_V_*	Vaccination supply rate	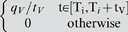 where *T_i_* are vaccination delivery times

Since it is unclear how many vaccinations will be required to eliminate the cancer, we will optimize the treatment strategy according to the following method:

1. **Imatinib**. Begin imatinib treatment at time 0 and continue treatment throughout immunotherapy.
2. **Timing**. For a given dosage, assume that there is only one vaccination and find the optimal timing such that the resulting minimum cancer concentration is as low as possible. This will determine the timing of the first vaccination.
3. **Pacing**. Continue delivering vaccinations of the same dosage at fixed time intervals until eliminating leukemia (according to the elimination criterion Equation 5, defined below).

At the end, we select the vaccination strategy that attains the lowest minimum leukemia concentration with the fewest vaccinations. We implement this optimization strategy, because it is more efficient than attempting to globally optimize several vaccinations of varying dosage and irregular time intervals at once. Indeed, it is a one-dimensional search problem, as opposed to a higher dimensional problem. For this assessment, we also assume that there are no mutations, i.e. *u* = 0. Results of relaxing this assumption are discussed below.

We numerically solve the system given by Equations 2–4 using the DDE solver ‘dde23’ from Matlab 7.0. For each run, we evaluate the solution up to day 400. We use parameter sets from Table 6 and examine vaccination dosages of 0.1 to 1 *k*/*µL*.

For each fixed dosage, we find optimal vaccination delivery times (up to a day), and our goal is to drive the cancer below 1 cell/6 *L*∼10^−10^ *k*/*µL*, i.e.
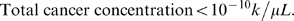
(5)


We assume that the criterion (Equation 5) represents cancer elimination. Since this model is a continuous deterministic system, in reality, Equation 2 never allows the cancer population to actually reach 0.

Using the aforementioned method of optimization, we optimize the timing of a series of vaccinations of varying dosages. We measure dosages in units of concentration (*k*/*µL*), referring to the average concentration of inactivated leukemia cells in the patient's body. Since the average person has about 6 L of blood, a vaccination of 1 *k*/*µL* corresponds to 6×10^9^ inactivated leukemia cells. For the parameters from Table 6 corresponding to P4, the optimal delivery times and minimum cancer concentrations for up to five vaccinations of varying dosages are given in Table 8.

**Table 8:** Vaccination strategies for P4.

Dose (*k*/*µL*), number of cells	Timing	Pacing	Number	log_10_ [Min cancer load]
0.1, 6.0×10^8^	233	10	5	−10.5
1.0, 6.0×10^9^	240	-	1	−10.4

For each dosage, the timing indicates the day on which the first vaccination is given, and the Pacing indicates the number of days between subsequent vaccinations. The Number indicates the number of vaccinations administered, and the final column indicates the base 10 logarithm of the minimum cancer concentration attained after the final vaccination. Values of less than 10^−10^ correspond to fewer than one cell in the body and denote cancer elimination.

On one hand, five vaccinations of dosage 0.1 *k*/*µL* can eliminate cancer in P4. On the other extreme, one vaccination of dosage 1.0 *k*/*µL* works as well. Depending on whether it is more important to eliminate cancer as fast as possible or to minimize the total dosage of irradiated cancer cells, different strategies may be preferable. Also, it may be advantageous to vary vaccination dosages over time and consider dosages below 0.1 *k*/*µL*, but a thorough analysis of this optimization problem lies beyond the scope of this paper.

**Figure 6:**
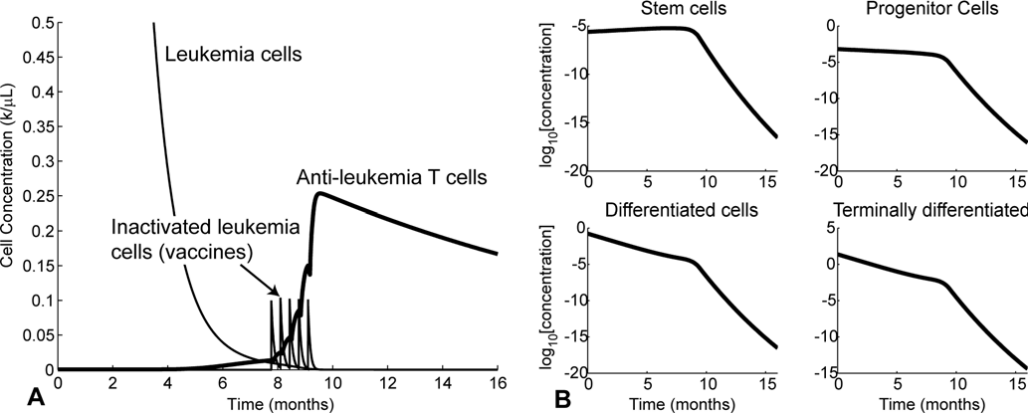
The treatment strategy in row 1 of Table 8. (A) Time evolution of cancer and T cell populations. Vaccinations are delivered on days 233, 243, 253, 263, and 273. (B) Time evolution of the four types of leukemia cells: stem cells (SC), progenitors (PC), differentiated cells (DC), and terminally differentiated cells (TC). Concentrations are shown on a logarithmic scale.

The time evolution of the cancer and T cell populations for the treatment strategy in row 1 of Table 8 are shown in Figure 6. Repeated stimulation of T cells by the five vaccinations causes the T cell level to multiply to over 10 times the T cell peak with imatinib alone in Figure 2B. As a result, the leukemia population is completely eliminated.

In addition, analogous tables of vaccination strategies for P1 and P12 are shown in Tables 9 and 10. The T cells of P1 and P12 seem to be less responsive than those of P4, since they require higher dosages to eliminate the leukemia cells.

**Table 9 pcbi-1000095-t009:** Vaccination strategies for P1.

Dose (*k*/*µL*), number of cells	Timing	Pacing	Number	log_10_ [Min cancer load]
0.1, 6.0×10^8^	202	5	12	−10.7
2.3, 6.0×10^10^	209	-	1	−10.2

**Table 10:** Vaccination strategies for P12.

Dose (*k*/*µL*), number of cells	Timing	Pacing	Number	log_10_ [Min cancer load]
0.1, 6.0×10^8^	195	4	11	−10.1
2.0, 1.2×10^10^	199	-	1	−10.4

In all cases, the first vaccinations are given before the peak of the T cell responses to boost the response. The peaks of the T cell populations fall between months 9 and 10 (see Figure 2), and in all cases, the first vaccinations fall around months 7 to 8. It is optimal to give the first vaccination before the T cell response begins to contract.

Thereafter, the following vaccinations serve to sustain the immune response over an extended time, so the gaps between these vaccinations depend on how long it takes for the previous vaccination to clear out of the system.

In the data of [8] there were 2 patients with no detectable immune response (P10 and P14). The leading hypothesis there was that the immune response was present but that it was below the detection level. In the mathematical model, low-level immune responses can be obtained when immune down-regulation from leukemic cells is high or when T cell division is low. In these cases, vaccinations still boost the immune response and eliminate leukemia, but dosages must be higher or they must be administered for longer periods. In addition, if the level of immune down-regulation from leukemia cells is very high, the timing of the first vaccination will be much later than for P1, P4, and P12, since it will take longer for the leukemia population to pass into the optimal load zone. Because these immune responses are too low to detect using ELISPOT, we do not have data points to fit the model. Hence, any vaccination strategy for P10, P14, or any of the other patients with no detectable response will be highly speculative. For such patients we forgo any quantitative conclusions and instead state that strategic vaccinations are likely to enhance any anti-leukemia T cell response, even when the anti-leukemia response is low.

### Importance of Timing and Pacing

The timing and pacing of the vaccination strategies are critical to the success of the outcome. For example, consider the alternative vaccination strategies in Tables 11, 12, and 13. These are deviations from the optimized vaccination strategies in Tables 8, 9, and 10, respectively.

**Table 11:** Alternative vaccination strategies for P1.

Dose (*k*/*µL*), number of cells	Timing	Pacing	Number	log_10_ [Min cancer load]
0.1, 6.0×10^8^	1–30	5	12	−3.3
	300	5	12	−6.2
	233	1	12	−8.0
	233	20	12	−5.4
2.3, 1.4×10^10^	1–30	-	1	−3.3
	300	-	1	−7.3

**Table 12 pcbi-1000095-t012:** Alternative vaccination strategies for P4.

Dose (*k*/*µL*), number of cells	Timing	Pacing	Number	log_10_ [Min cancer load]
0.1, 6.0×10^8^	1–30	10	5	−3.2
	300	10	5	−7.6
	233	1	5	−6.7
	233	20	5	−9.4
1.0, 1.2×10^10^	1–30	-	1	−3.2
	300	-	1	−8.6

**Table 13:** Alternative vaccination strategies for P12.

Dose (*k*/*µL*), number of cells	Timing	Pacing	Number	log_10_ [Min cancer load]
0.1, 6.0×10^8^	1–30	4	11	−3.1
	300	4	11	−5.9
	195	1	11	−8.3
	195	20	11	−5.9
2.0, 1.2×10^10^	1–30	-	1	−3.1
	300	-	1	−6.7

Note that if vaccinations are initiated within 30 days of the start of imatinib treatment, the effect of the vaccinations is insignificant. There is hardly any anti-leukemia immune response, and the decline in the leukemia population is mainly due to natural death under imatinib. This happens since within the first 30 days, the leukemia population is still well above the optimal load zone, and in general, vaccinations are ineffective when the leukemic load is above the optimal load zone (where the immune suppression is too strong). However, once the leukemic population is sufficiently low, the optimal load is no longer an issue, since inactivated leukemic cells that have no immunosuppressive effects are used for vaccinations.

On the other hand, administering vaccinations too late (e.g. at 300 days) is not entirely ineffective, since leukemia has already passed into remission and no longer exerts a great immuno-suppressive effect. However, 300 days after the start of treatment, the initial anti-leukemia T cell response has started to decline, so the response to vaccination is not as strong. By considering early and late vaccinations, we see that optimizing vaccination delivery times depends on a balance between minimizing the immuno-suppressive effect of leukemia and maximizing the available anti-leukemia T cells to respond to the stimulus.

In the same way, in multiple vaccination strategies, there is an optimal pacing between vaccinations that will optimally maintain the immune stimulation over time. As we can see from Tables 11–13, excessively low and high intervals of 1 and 20 days lead to less effective vaccination strategies. Hence, by fitting patient data and modeling various vaccination strategies, we can predict the most effective ways to utilize the available resources for a maximal impact.

### Overloading Vaccination Strategies and Sensitivity to *d_T_*, *c_n_*, and *n*

In general, the vaccination strategies for each patient will still work if T cells die at slower rates, if the immune-suppression is lower, or if T cells divide more after stimulation. These scenarios correspond to decreasing *d_T_*, decreasing *c_n_*, and increasing *n*. In these cases, the vaccination strategies should not only continue to work, but should also become more effective.

However, our optimization strategy seeks to find the lowest dosage or smallest number of vaccinations necessary to eliminate cancer. Hence, these strategies are sensitive to underestimates of *d_T_*, underestimates of *c_n_*, and overestimates of *n*. To allow a buffer for a more robust vaccination plan, we can develop an optimal strategy for overloading the optimal vaccination strategies. For example, we can give P4 one vaccination of dosage 2.0 *k*/*µL* rather than 1.0 *k*/*µL*. Alternatively, we can give P4 10 vaccinations of dosage 0.1 *k*/*µL* instead of 5 vaccinations. This will allow the vaccinations to be much more reliable and robust to errors in the parameter estimates.

A full treatment on the optimal way to overload a vaccination strategy leads to more complex optimization problems, which we leave for future work. However, in Tables 14–16, we consider the effect of doubling the vaccination dosages or the number of vaccinations for P1, P4, and P12. For each overloaded vaccination strategy, we report the amount that *c_n_* and *n* can vary from their original values without rendering the strategy ineffective. For convenience, variabilities are reported as ± (some percentage), but in reality, these variabilities only correspond to upper bounds for *c_n_* and lower bounds of *n*. Since the parameter *d_T_* is more readily measured from T cell decay rates, we exclude it from the sensitivity analysis.

**Table 14:** Overloaded vaccination strategies for P1.

Dose (*k*/*µL*)	Timing	Pacing	Number	Allowed variability for *c_n_* and *n*
2×0.1	202	5	12	±10%
0.1	202	5	2×12	±10%
2×2.3	209	-	1	±4%

**Table 15 pcbi-1000095-t015:** Overloaded vaccination strategies for P4.

Dose (*k*/*µL*)	Timing	Pacing	Number	Allowed variability for *c_n_* and *n*
2×0.1	233	10	5	±10%
0.1	233	10	2×5	±12%
2×1.0	240	-	1	±5%

**Table 16:** Overloaded vaccination strategies for P12.

Dose (*k*/*µL*)	Timing	Pacing	Number	Allowed variability for *c_n_* and *n*
2×0.1	195	4	11	±7%
0.1	195	4	2×11	±8%
2×2.0	199	-	1	±4%

From this preliminary analysis, it appears that doubling is more effective for strategies consisting of vaccine low dosages administered multiple times. It is unclear whether it is more effective to double the dosages or to double the number of times vaccinations are administered, since the relative efficacies of each approach vary from patient to patient. In any case, overloading vaccinations seems to be an effective method for increasing the robustness of vaccination strategies against uncertainties in parameter values.

### Sensitivity Analysis

The analysis in the previous sections focused on three particular patients and proposed three different treatment strategies for each case. However, to extend out findings to a general approach, we would like to examine which scenarios favor one vaccination regime over another. Indeed, from Tables 8–10, we notice that patient P4 requires a much lower vaccination dosage than patients P1 and P12 to obtain an adequate immune response. This observation implies that the immune response of P4 is more active than the immune response of P1 and P12. We would thus like to understand which measurable parameters and initial conditions can be correlated to the effectiveness of vaccination therapy. Furthermore, we would like to understand how the treatment strategies can be adapted to the individual patients based on the measured strength of their immune responses.

To study the correlation between parameters and the effectiveness of proposed vaccination strategies, we apply the Latin Hypercube sampling (LHS) method [20]. This method provides means of simultaneously sampling a wide range of dynamical parameters and is useful for statistically determining which parameters correlate highly to certain desired outcomes. LHS involves numerically simulating the model multiple times with randomly sampled sets of parameters. The samples are chosen such that each parameter is well distributed over its range of possible values.

For each LHS simulation, we test one vaccination strategy over a range of 500 randomly sampled parameter sets. The parameters are sampled uniformly over the ranges indicated in Table 17. As indicated in the table, we vary every parameter and initial condition except the decay rate of dead cancer cells (which are used only in vaccinations).

**Table 17:** Parameter ranges to be used for Latin Hypercube sampling.

	Description	Estimate	Range	PPMC	SROC
*λ*	fractional adjustment constant	0.75	0.5 to 1	−0.2152	−0.1395
*d*_0_	SC death rate	0.003 *λ*/day	±25%	−0.0354	−0.0123
*d*_1_	PC death rate	0.008*λ*	±25%	−0.0643	−0.0066
*d*_2_	DC death rate	0.05*λ*	±25%	−0.1497	−0.0130
*d*_3_	TC death rate	*λ*	±25%	0.0206	0.0080
*r_y_*	Growth rate for nonresistant cells without imatinib treatment	0.008/day	±25%	0.0242	0.0174
*a_y_*		1.6	±25%	−0.0366	−0.0259
*b_y_*		10	±25%	0.0372	0.0087
*c_y_*		100	±25%	0.0419	0.0411
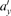	Rates during imatinib treatment	*a_y_*/100	Same as *a_y_*/100	-	-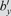		*b_y_*/750	Same as *b_y_*/750	-	-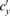		*c_y_*	Same as *c_y_*	-	-
*r_z_*	Growth rate for resistant cells	0.023/day	±25%	0.0036	0.0158
*a_z_*		*a_y_*	Same as *a_y_*	-	-
*a_z_*		*b_y_*	Same as *b_y_*	-	-
*c_z_*		*c_y_*	Same as *c_y_*	-	-
*u*	Mutation rate per division	4×10^−8^/division	±100%	−0.0156	0.0252
*k*	Kinetic coefficient	1(*k*/*µL*)^−1^ day^−1^	±25%	−0.1241	−0.1287
*p*_0_	Prob. T cell engages cancer cell	0.8	±25%	−0.1328	−0.1606
*q_C_*	Prob. cancer cell dies from encounter	0.75	±25%	0.0084	0.0105
*q_T_*	Prob. T cell survives encounter	0.5	±25%	−0.0947	−0.1419
*τ*	Duration of one T cell division	1 day	12–24 hrs	0.0676	0.0301
*N*	Avg no. of cell divisions	1.17 to 2.2	1 to 3	−0.4681	−0.6889
*d_T_*	T cell death rate	1–7×10^−3^/day	1E-3 to 1E-2	0.1786	0.2523
*d_V_*	Inactivated leukemia cell decay rate	0.35/day	Not varied	-	-
*s_T_*	T cell supply rate	1E-5 to 1E-6 *k*/*µL*/day	1E-5 to 1E-6	−0.0412	−0.0557
*c_n_*	Decay rate of immune responsivity	0.8 to 7/day	0 to 10	0.1785	0.2623
*C*(0)	Pre-treatment cancer load	23.1–116.8 *k*/*µL*	20 to 200	0.1819	0.0717

Also, shown are correlations between parameters and minimum cancer concentrations. Correlation coefficients are as follows: Pearson product-moment correlation (PPMC), Spearman rank-order correlation (SROC).

From Tables 8–10, we see that the optimal start times for our vaccination strategies are between around 200 and 240. Furthermore, the optimal pacing between vaccinations is between around 5 and 10 days. Using these numbers as guides, we test several variations of vaccination strategies with start times at either 200 or 240 days and pacings of 5, 10, 20, or 40 days. Furthermore, we try vaccination dosages of 0.1 and 0.2 *k*/*µL*. The results are shown in Table 18. In this table, alongside the tested vaccination strategies, we include the fraction of 500 LHS samples that result in cancer elimination. The first eight strategies consist of five vaccinations with dosage 0.1 *k*/*µL*. Hence, these strategies require a total dosage of 0.5 *k*/*µL*. If we assume that the average person has 6 L of blood, this total dosage corresponds to 0.5 *k*/*µL*×6×10^6^ *µL* = 3×10^9^ leukemia cells.

**Table 18:** Tested vaccination strategies and fraction of LHS samples that result in successful treatments.

Total dosage (*k*/*µL*)	Dosage (*k*/*µL*)	Schedule	(Number of successes)/500
0.5	0.1	200, 205, 210, 215, 220	0.474
	0.1	200, 210, 220, 230, 240	0.490
	0.1	200, 220, 240, 260, 280	0.502
	0.1	200, 240, 280, 320, 360	0.488
	0.1	240, 245, 250, 255, 260	0.510
	0.1	240, 250, 260, 270, 280	0.514
	0.1	240, 260, 280, 300, 320	0.520
	0.1	240, 280, 320, 360, 400	0.472
1.0	0.1	200, 205, 210, 215, 220, 225, 230, 235, 240, 245	0.578
	0.1	200, 210, 220, 230, 240, 250, 260, 270, 280, 290	0.622
	0.1	200, 220, 240, 260, 280, 300, 320, 340, 360, 380	0.610
	0.1	240, 245, 250, 255, 260, 265, 270, 275, 280, 285	0.630
	0.1	240, 250, 260, 270, 280, 290, 300, 310, 320, 330	0.642
	0.1	240, 260, 280, 300, 320, 340, 360, 380, 400, 420	0.616
	0.1	200, 210, 220, 230, 240	0.570
	0.1	240, 250, 260, 270, 280	0.624
	0.1	200	0.482
	0.1	240	0.532

(Assuming the average person has 6 L of blood, the total number of leukemia cells needed for each vaccination strategy is (Total dosage)×6×10^6^ *µL*).

Among these first eight vaccination strategies with a total dosage of 0.5 *k*/*µL*, we see that vaccination strategies that start on day 240 are more effective than those that start earlier on day 200. In addition, it seems that a pacing of 20 days between vaccinations performs better than the alternative pacings of 5, 10, and 40 days. Observing the trend in the eight tested strategies, it seems that the optimal pacing would fall between 10 and 40 days.

Among the ten strategies with total dosage 1.0 *k*/*µL*, it seems again that starting vaccinations on day 240 is better than starting too early on day 200. Furthermore, it seems that spreading the vaccinations out among ten small doses is more effective than grouping them into five larger doses or one very large dose.

In this work we only examined a limited set of possible vaccination strategies. Indeed, there is no reason to require that all vaccinations will be of the same size or that the pacing will remain uniform. However, generalizing our survey of possible strategies poses a challenging optimization problem that is beyond the scope of the current paper.

On the other hand, LHS sampling allows us to determine the statistical correlations between the treatment outcomes and a wide range of model parameters. For each LHS simulation, we measure the correlations between the varied parameters and two indices: the minimum cancer concentration attained during the course of simulation (600 days) and the success of the treatment strategy. For our correlations, we use the Pearson and Spearman rank-order coefficients, and consider a treatment to be successful if it causes the cancer concentration to drop below 10^−10^ *k*/*µL*, which is approximately the concentration of half a cancer cell in the blood.

**Figure 7:**
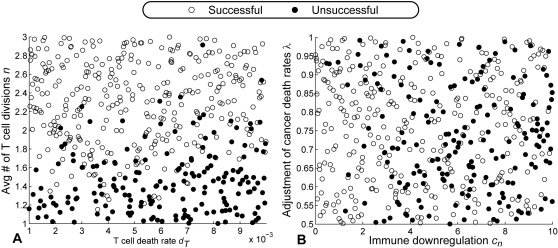
A sensitivity study. (A) Scatter plot of LHS simulation results with respect to the T cell death rate, *d_T_*, and the average number of T cell divisions upon stimulation, *n*. As apparent from the figure, the treatment outcomes are highly correlated to the values of *n*. (B) Scatter plot of LHS simulation results with respect to the immune downregulation, *c_n_*, and the fraction adjustment for cancer death rates, *λ*.

In Table 17, we show the Pearson and Spearman rank-order coefficients for the fifth vaccine with total dosage 0.1 *k*/*µL*. (This is the strategy with dosage 0.1 *k*/*µL* per vaccination with vaccinations scheduled every 10 days between day 240 and 330). Figure 7 shows scatter plots of simulation results for this vaccination strategy with respect to (*d_T_*, *n*) and (*c_n_*, *λ*). The correlations obtained from all other tested strategies are comparable.

We see from the correlation table that the most sensitive parameter is the average number of T cell divisions per stimulation, *n*. This result makes sense because *n* is a direct measurement of the inclination of T cells to proliferate. More responsive T cells will perform much better under vaccinations. This observation implies that the T cell activity of a patient, and especially its inclination to proliferate, should be measured (probably *in vitro*) before and during the imatinib treatment to gauge the intensity of the vaccination treatment that is needed to ensure sufficient T cell expansion.

There are three additional parameters that have some influence on the outcome. These are the T cell death rate, *d_T_*, the level of immune downregulation, *c_n_*, and the fractional adjustment constant for cancer death rates, *λ*. The T cell death rate is relevant, because T cells that die faster require a prolonged stimulation to remain at effective levels. This is one reason why spreading vaccinations out across many smaller doses seems largely more effective than combining vaccinations into larger doses. The latter strategy would only be preferable if the average T cell death rate is low enough to allow the T cell response to persist for a long time without stimulus. The parameter *λ* directly affects the time it takes for cancer to enter remission. If *λ* is low, it means that imatinib is less effective, so it takes longer for a patient to enter remission. Furthermore, the level of immune downregulation, *c_n_*, affects the time that downregulation becomes low enough to make the vaccinations effective. The combined effects of *λ* and *c_n_* affect the optimal start time of vaccination treatment. Low *λ* and high *c_n_* would require the start time of the vaccination to be postponed until downregulatory effects have diminished. While it may be difficult to measure these parameters directly, the ambient level of immune downregulation can be deduced by tracking T cell activity during the course of imatinib treatment.

All the parameters other than the four discussed above have little influence on the outcome of the treatment. We especially point out that the mutation rate per cell division, *u*, and the initial leukemia load, *C*(0), are among the irrelevant parameters. While the initial loads will vary by at most a factor of three or so, remission time is much more strongly affected by the exponential decay rate of cancer under imatinib, which pertains more to the parameter *λ* than anything else. Ultimately, the sensitivity analysis shows that *n*, which measures the T cell responsivity upon stimulation, is the key parameter to predicting the effectiveness of vaccination strategies. As a potential clinical application, relevant dynamical parameters can be measured *in vitro* before and during the course of treatment to devise or adjust a vaccination strategy to optimize anti-leukemia T cell stimulation.

## Discussion

Among all mathematical models of CML, our approach is unique in the sense that the experimentally observed anti-leukemia immune response is incorporated into the model. With the addition of the T cell response in our model, persistence of anti-leukemia T cells even at low levels seems to prevent the leukemia from relapsing (for at least 50 months). We therefore hypothesize that anti-leukemia T cells responses may help maintain remission under imatinib therapy. Therapy with imatinib (and other targeted therapies being developed) has the advantage to target leukemic cells more selectively than non-specific therapies such as chemotherapy and radiation. As such, host immune function, including antigen presentation, may be restored more rapidly than after chemotherapy, due to alleviation of leukemia-induced immune suppression. Importantly, normalization of host immune function, while leukemia antigens are still present, may optimally drive anti-leukemia immune responses.

Our model suggests that the balance between immune down-regulation and T cell stimulation by leukemic cells determines the effectiveness of the anti-leukemia T cell response. Studying the optimal level of stimulation led us to define the novel concept of an “optimal load zone” as the range of leukemic cell concentrations where the T cell stimulation rate is optimal. In general, imatinib causes the leukemic cell population to fall into the optimal load zone, stimulating a T cell response most efficiently and to the highest amount before it drops out of this zone. At a certain threshold below the optimal load zone, leukemic cells become essentially invisible to T cells due to low interaction rates, and the immune response contracts. At this point, one would need exogenous stimulation to maintain T cell proliferation.

This led us to hypothesize that cryopreserved autologous leukemic cells, inactivated by irradiation, may be given to patients in remission as vaccines to enhance T cells responses. To study this approach, we added inactivated leukemic cells (unable to proliferate or exert immune suppression) to our model. A strategy of immunotherapy and imatinib treatment for each patient was constructed using an optimization algorithm. Our model predicts that the timing and pacing of the vaccinations are crucial.

Although vaccination optimizations are presented for particular patients, it may be possible to devise a more general strategy that works most of the time. Furthermore, the parameter fitting can be more refined to consider several likely parameter sets, and the optimization problem can be expanded to consider variable vaccination dosages *q_V_*_,1_, *q_V_*_,2_,…, *q_V,n_* under the constraint 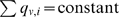.

Another question is whether the effects of vaccination can be clinically observed. Since most leukemia patients taking imatinib undergo cytogenetic remission, but not molecular remission (P.P.L., unpublished data), it is possible to observe whether vaccinations can further drive the leukemia to molecular remission. The thresholds for cytogenetic and molecular remission are 10^8^ and 10^6^ leukemia cells in the body, respectively. Assuming that an average person has 6 L of blood, these counts correspond to leukemia concentrations around 10^−2^ and 10^−4^ *k*/*µL*, respectively. Thus, the model predicts that a series of vaccinations will not only drive the leukemia population below the molecular remission level, but will actually drive it to extinction.

To clinically implement these treatments, one would also need to have a criterion for starting the vaccinations. From the model, we observed that vaccinations are best administered just prior to the peak of the T cell response; however, in practice, it may be hard to determine the T cell peak times. We observe that for all patients, the T cell peaks occurred around 10 months after starting the imatinib treatment, while they entered complete and major cytogenetic remissions a few months earlier. Determining whether there is a correlation between remission times and T cell peak times will prove useful in carrying out treatments, and may be the goal of future studies. Such a study would require simultaneously measuring the T cell and the leukemia levels over time, perhaps at the molecular level.

An important issue is whether stem cells are immunologically privileged. In principle, T cells are known to have the capacity of killing leukemia stem cells as evidenced by the success of allogeneic bone marrow transplants. It is unknown whether the autologous immune response can produce similar results. It is also possible that leukemia cells may down-regulate target molecules for the anti-leukemia T cells. However, this rate is probably much slower than the rate of acquiring imatinib resistance. In any case, even if stem cells or mutated leukemia cells were immunologically privileged, what we propose may still substantially delay the leukemia relapse. Indeed, [21] and [22] show that an active immune response in conjunction with imatinib plays a significant role in the elimination of leukemia. These papers describe experiments in which imatinib was given to CML patients who relapsed after allogenic bone marrow transplants, resulting in sustained remission.

We also observe that the more demanding vaccination strategies for each patient P1, P4, and P12 require total doses of 2.3 *k*/*µL*, 1.0 *k*/*µL*, and 2.0 *k*/*µL*, respectively. These samples can be obtained from 6 L×2.3/73 = 190 *mL*, 6 L×1.0/23.1 = 160 *mL*, 6 L×2.0/116.8 = 100 *mL* of pre-treatment blood from P1, P4, and P12, respectively. Since we are only interested in collecting leukemia cells prior to imatinib treatment, these samples can be gathered by filtering the white blood cell component of the patient's blood. For reference, we point out that one US pint is about 500 ml, and this quantity of whole blood is routinely collected from healthy individuals.

An issue that was not investigated directly in this study is the functional form of immune downregulation. In our model, we chose to use the form 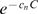, i.e., an exponential decay. It will be difficult to conduct a sensitivity analysis with respect to the function form. However, as implied by the previous discussion, the functional form does not greatly affect outcome of vaccination strategies as long as there is very low residual downregulation after cancer remission. In other words, when leukemia drops below remission, immune cells are no longer effectively downregulated. Since downregulation is usually hypothesized to be the result of contact-dependent mechanisms or suppression by negative cytokine signaling, it follows that the effects of downregulation will most likely disappear or at least become greatly reduced once the source of downregulation is removed.

As a final point, we note that in the Michor model leukemia relapses at 15 to 24 months despite continued imatinib therapy with the Michor model [5]. This results from imatinib's inability to control leukemic stem cells - a conclusion of this previous work [5]. However, this contradicts clinical observations in imatinib-treated patients [23], who generally remain in remission well beyond 30–40 months. With the addition of the T cell response in our model, persistence of anti-leukemia T cells even at low levels prevented leukemia from relapsing for up to 50 months. We therefore hypothesize that anti-leukemia T cell responses may help maintain remission under imatinib therapy. Therapy with imatinib (and other targeted therapies being developed) has the advantage to target leukemic cells more selectively than non-specific therapies such as chemotherapy and radiation [24]. As such, host immune function may be restored more rapidly than after chemotherapy, due to alleviation of leukemia-induced immune suppression. Importantly, normalization of host immune function, while leukemia antigens are still present, may optimally drive anti-leukemia immune responses. It should be noted that imatinib was shown to have some immunomodulatory activity in a mouse arthritis model [25].

An alternative model of CML dynamics was recently published by Roeder *et al.* [6]. In this model, stem cells exist in non-proliferating or proliferating states. The likelihood for a stem cell to proliferate and differentiate or to return to dormancy is determined by an internal mechanism, called the affinity function. After imatinib treatment, which in this model can target proliferating but not non-proliferating stem cells, most remaining stem cells are dormant, resulting in a much longer remission and a slower relapse than the Michor model [5]. In view of our present work, it is important to note that the model in [6] does not include the immune response. However, quantitative data for the non-proliferating or proliferating states are not available. In either case, the models do not significantly diverge for the first few years and our analysis focuses within this time period when the anti-leukemia immune response is still active. Hence, the anti-leukemia immune response that we observed experimentally and modeled is consistent with both models.

### Conclusions

The approach presented in this paper accounts for the role of the anti-leukemia specific immune response in the dynamics of CML. By combining experimental data and mathematical models we demonstrate that persistence of anti-leukemia T cells even at low levels seems to prevent the leukemia from relapsing (for at least 50 months). Consequently, we hypothesize that anti-leukemia T cells responses may help maintain remission under imatinib therapy.

The mathematical model together with the experimental data of [8] imply that there may be a feasible, low risk, clinical approach to enhancing the effects of imatinib treatment. These conclusions rest on the hypotheses that imatinib induces an innate immune response and that the patient's immune system functions alongside imatinib to sustain cytogenetic remission for up to several years.

The mathematical modeling of experimental data provides insights, suggesting that these responses may play a critical role in maintaining remission. Our model suggests that properly timed vaccinations with autologous leukemic cells, in combination with imatinib, can sustain the T cell response and potentially eradicate leukemic cells. It also shows that vaccinations must be optimally timed in relation to host anti-leukemia T cell responses. A key assumption in the model is that anti-leukemia T cells can target all leukemic cells (including stem cells and cells that develop resistance to imatinib). Such an assumption is supported by the graft-versus-leukemia response of allogeneic stem cell transplantation [26],[27] suggesting that leukemic stem cells can be eliminated by the immune response. Resistance to imatinib, such as via abl mutations, could render a leukemic cell even more susceptible to immune targeting. Even if this is not the case, the proposed therapeutic strategy could still potentially result with a substantial increase of the expected time to a relapse. Combining imatinib with optimally timed vaccinations could lead to a potential cure of CML. While cancer vaccines is not a new concept, the importance of optimal timing of vaccinations in relation to the underlying endogenous immune response (which the vaccine attempts to boost) is novel and not previously suggested in the field of cancer immunotherapy. This approach may be transferable to other cancers, as other molecular targeted therapies become available.

While it is still too early to begin human clinical trials with our novel immunotherapy strategies, our immediate goal is to refine and validate our model predictions with additional patient measurements, and only then propose a clinical trial. There is still no good animal model of CML to validate our model predictions or test various vaccination conditions. As such, we are continuing to analyze samples from additional patients - at higher resolution time points guided by our results thus far. We will particularly focus on patients that relapse on imatinib to study their immune responses before, during, and after the relapse period. Such patients are now being put on next generation molecular targeted drugs such as dasatinib, which will bring 80% of patients with imatinib-resistant leukemia back into remission. We will analyze the immune responses in these patients. At all time points, we will obtain accurate measurements of the leukemia load via real-time PCR. This will allow us to validate our predictions for the optimal load zone.
